# Risk factors for lethal outcome in patients with delirium tremens - psychiatrist's perspective: a nested case-control study

**DOI:** 10.1186/1744-859X-12-39

**Published:** 2013-12-02

**Authors:** Dragana Ignjatovic-Ristic, Nemanja Rancic, Slobodan Novokmet, Slobodan Jankovic, Srdjan Stefanovic

**Affiliations:** 1 Psychiatric Clinic, Clinical Center Kragujevac, Faculty of Medical Sciences, University of Kragujevac, Kragujevac 34000, Serbia; 2 Center for Clinical Pharmacology, Medical Faculty Military Medical Academy, University of Defence, Crnotravska 17, Belgrade 11000, Serbia; 3 Department of Pharmacy, Faculty of Medical Sciences, University of Kragujevac, Kragujevac 34000, Serbia; 4 Department of Pharmacology, Faculty of Medical Sciences, University of Kragujevac, Kragujevac 34000, Serbia

**Keywords:** Delirium tremens, Mortality, Serum potassium level, Number of hospitalization, Length of stay (duration of hospitalization)

## Abstract

**Background:**

The aim was to identify potential risk factors for lethal outcome in patients with delirium tremens (DT) treated in the psychiatric setting.

**Methods:**

In a nested case-control study, a total of 190 medical records of patients with DT hospitalized at the Psychiatric Clinic in Serbia between 2002 and 2011 were reviewed and analyzed. The characteristics of patients who died (cases) were compared with those who survived (controls). For each case, two controls (matched for age, gender, and year of hospitalization) were randomly chosen.

**Results:**

Significant differences between cases and controls were found for serum potassium levels (*p* < 0.001), the number of hospitalizations (*p* < 0.001), and duration of hospitalization (*p* < 0.001). A significant association with lethal outcome was found for serum potassium levels even in the normal range (adjusted odds ratio 40.52; 95% CI 1.20, >1,000.00; *p* = 0.004).

**Conclusions:**

Even though the number and duration of psychiatric hospitalizations were identified as factors determining survival after admission for DT, only serum potassium levels were found to be significant. Patients with an increase in potassium (or absence of hypokalemia) may require more intensive treatment. Monitoring of serum levels of potassium is important not only as an indicator for replacement but also as an indicator of maladaptation.

## Background

Delirium tremens (DT) is the severest form of alcohol withdrawal and is recorded in approximately 5% to 24% of patients [1-5]. Over the last few decades, the mortality rate for patients with DT has fallen from 35% to 2%, and this has been largely attributed to improved diagnostic and pharmacological measures [2,6-11]. Several studies have identified risk factors for the occurrence of DT [5,12-18], but there has been little research into the risk factors for mortality among patients with DT [3,6,10,18,19]. The factors that predict a fatal outcome in patients with DT remain unclear, and DT is still categorized as an emergency [2,3,5,20]. The early recognition of risk factors for DT mortality in patients will facilitate the introduction of preventative measures to decrease the likelihood of a fatal outcome [3,21]. The danger of a fatal outcome in the psychiatric setting, where DT patients are first seen or treated, additionally highlights the importance of early recognition and treatment of DT.

The aim of the study was to identify important risk factors for fatal outcome in patients with DT who presented for admission at the Psychiatric Clinic of the Clinical Center ‘Kragujevac’ in Serbia.

## Methods

### Study design

The study design is a nested case-control study.

### Study population

The study was conducted using data obtained for all patients with DT (*n* = 190) who were hospitalized at the Psychiatric Clinic of the Clinical Center ‘Kragujevac’ in Serbia, during the 10-year period (2002–2011). Patients were included in the study if they were 18 years of age and above and if they fulfilled the International Classification of Diseases (ICD-10) criteria for DT [22,23]. Patients with incomplete data sets were excluded. Cases (*n* = 14) were defined as patients who had died from DT. Two controls (*n* = 28) were identified for each case; matched for gender, age, and calendar year in which they were treated; and randomly selected from the patients with DT who had been successfully treated and discharged alive. All patients were treated according to the standardized therapeutic protocol at the clinic. Ethical approval was obtained from the ethical committee of Clinical Center ‘Kragujevac’ and the principles of Good Clinical Practice were strictly followed.

### Study protocol

For all patients, we collected demographic data (age, gender, marital and educational status) and variables relating to alcohol consumption (heredity factors, duration of alcohol consumption). Serum potassium levels, aspartate aminotransferase/alanine aminotransferase levels (AST/ALT), and level of creatine phosphokinase (CPK) in serum were measured in each patient on the day of admission. Data on further potential risk factors for a fatal outcome were also recorded, namely, existence of liver lesions (clinical diagnosis by gastroenterologist, without ultrasound confirmation), epileptic seizure during the 24 to 48 h following admission (without previous epilepsy), transfer to another clinical department, and number and duration of previous hospitalizations for alcohol-related health problems at this clinic.

### Statistical analysis

Statistical analysis was carried out using the statistical software IBM SPSS version 19. Continuous variables were summarized as means (M) and standard deviations (SDs). Categorical variables were presented as the frequency of exposure (%) for each risk factor. For categorical variables, the frequency of exposure in cases was compared to that of controls using chi-square test or Fisher's exact test (*χ*^2^) (where the frequency in a category was small). Continuous variables were compared using Student's *t* test (*t*) for independent samples and the Mann-Whitney test (*U*). Normality of the data was assessed using the Kolmogorov-Smirnov test. The association between potential risk factors and lethal outcome was evaluated using binary logistic regression, expressing the strength of association by crude and adjusted odds ratio (OR) with 95% confidence intervals (CI). All significance tests were performed using a two-sided significance level of 5% (*p* < 0.05). The data were analyzed unmatched due to the small sample size.

## Results

**Figure 1:**
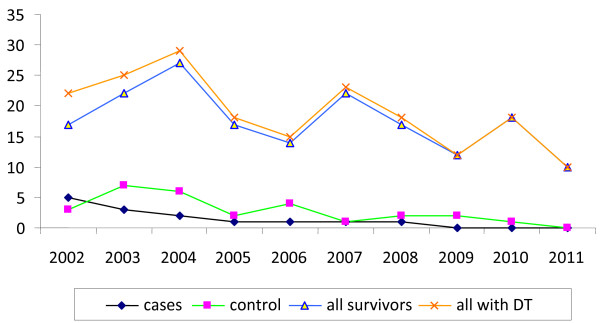
Number of patients per year in groups (cases, controls, all survivors, and all patients with DT).

In total, 190 (189 males, 1 female) patients satisfying the study criteria were identified during the study period. Of these, 14 (7.37%) patients died (cases). The mean age of all studied patients was 51.39 ± 9.82 years. Only two variables were independently associated with a fatal outcome. They were age and potassium levels in serum. Patients in the deceased group were significantly older (*p* = 0.037) and showed significantly higher serum potassium levels (*p* < 0.001); however, the levels were still within the normal range (Figure 1 and Table 1).

**Table 1:** Baseline characteristics of participants

	**Cases**	**Controls**	**Case/controls statistics**	**All survivors**	**All patients (cases + all survivors)**
Sex					
Male	14	28		175 (99.4%)	189 (99.5%)
Female	-	-		1 (0.6%)	1 (0.5%)
Age M ± SD	56.64 ± 11.34	56.39 ± 9.51	*t*(40) = −0.075; *p* = 0.940	50.97 ± 9.60	51.39 ± 9.82
Death					
No	-	28		176	176 (92.6%)
Yes	14	-		-	14 (7.4%)
Heredity					
No	11 (78.6%)	19 (67.9%)	*χ*^2^(1) = 0.131; *p* = 0.717	106 (60.2%)	116 (61.1%)
Yes	3 (21.4%)	9 (32.1%)		48 (27.3%)	51 (26.8%)
Missing	-	-		23 (12.5%)	23 (12.1%)
Hospitalization					
> 1	11 (78.6%)	6 (21.4%)	*χ*^2^(1) = 12.649; *p* = 0.001*	108 (61.4%)	119 (62.6%)
First	3 (21.4%)	22 (78.6%)		60 (34.1%)	63 (33.2%)
Missing	-	-		8 (4.5%)	8 (4.2%)
Duration of treatment in days M ± SD	4.29 ± 3.77	17.89 ± 11.90	*U* = 21.000; *p* = 0.000*	16.34 ± 8.39	15.43 ± 8.73
Marital status					
Not married	8 (57.1%)	20 (71.4%)	*χ*^2^(1) = 0.857; *p* = 0.355	58 (33.0%)	66 (34.7%)
Married/widower	6 (42.9%)	8 (28.6%)		104 (59.1%)	110 (57.9%)
Missing	-	-		14 (8.0%)	14 (7.4%)
Professional qualifications					
High	6 (42.9%)	16 (57.1%)	*χ*^2^(1) = 0.764; *p* = 0.382	53 (30.1%)	59 (31.1%)
Low	8 (57.1%)	12 (42.9%)		107 (60.8%)	115 (60.5%)
Missing	-	-		16 (9.1%)	16 (8.4%)
Duration of alcohol consumption in years M ± SD	19.64 ± 10.02	20.39 ± 12.20	*U* = 193.500; *p* = 0.946	17.41 ± 8.51	17.59 ± 8.63
K^+^ in serum M ± SD	4.50 ± 0.71	3.74 ± 0.59	*t*(40) = −3.661; *p* = 0.001*	3.87 ± 1.62	3.92 ± 1.58
AST/ALT ratio M ± SD	2.04 ± 1.43	1.80 ± 0.77	*t*(40) = −0.600; *p* = 0.557	2.05 ± 2.06	2.05 ± 2.01
CPK in serum M ± SD	2,910.79 ± 7,323.98	1,421.39 ± 1,841.46	*U* = 180.000; *p* = 0.669	1,413.82 ± 2,693.66	1,555.43 ± 3,391.42
Epileptic status					
No	11 (78.6%)	22 (78.6%)	*χ*^2^(1) = 0.000; *p* = 1.000	117 (66.5%)	128 (67.4%)
Yes	3 (21.4%)	6 (21.4%)		56 (31.8%)	59 (31.1%)
Missing	-	-		3 (1.7%)	3 (1.6%)
Liver damage					
No	10 (71.4%)	18 (64.3%)	*χ*^2^(1) = 0.214; *p* = 0.643	129 (73.3%)	139 (73.2%)
Yes	4 (28.6%)	10 (35.7%)		43 (24.4%)	47 (24.7%)
Missing	-	-		4 (2.3%)	4 (2.1%)
Transfer to another department					
No	13 (92.9%)	24 (85.7%)	*χ*^2^(1) = 0.454; *p* = 0.500	145 (82.4%)	158 (83.2%)
Yes	1 (7.1%)	4 (14.3%)		27 (15.3%)	28 (14.7%)
Missing	-	-		4 (2.3%)	4 (2.1%)

*p < 0.05 (significant difference); K^+^, potassium in serum; AST/ALT, aspartate aminotransferase/alanine aminotransferase; CPK, creatine phosphokinase; M, mean; SD, standard deviation.

### Cases and controls

Baseline characteristics for cases and controls are presented in Table 1. Serum potassium levels were significantly (*p* < 0.001) higher in cases even in the normal range (4.50 ± 0.71 mmol/l) compared to controls (3.74 ± 0.59 mmol/l). Cases also had a significantly greater number of hospitalizations (*p* < 0.001) and longer durations of hospitalization (*p* < 0.001). The multivariate analysis, using binary logistic regression analysis with adjustment for the risk factors, is summarized in Table 2. There was an independent significant association between lethal outcome and serum potassium level (OR_adjusted_ 40.52; 95% CI 1.20, >1,000.00; *p* = 0.004).

**Table 2:** Crude and adjusted odds ratios of the risk factors for mortality in DT

**Variables**	**Adjusted OR (95% CI)**	***p***	**Crude OR (95% CI)**	***p***
Age	1.49 (0.88, 2.54)	0.141	1.00 (0.94, 1.07)	0.939
Hospitalization	0.014 (0.00, 1.98)	0.091	1.35 (0.63, 2.82)	0.001
Marital status	3.40 (0.12, 99.96)	0.479	1.88 (0.49, 7.15)	0.357
Education	>100.00 (0.04, >1,000.00)	0.193	1.78 (0.49, 6.50)	0.384
Duration of consumption	0.63 (0.35, 1.12)	0.116	0.99 (0.94, 1.05)	0.839
Potassium in serum	40.52 (1.20, >1,000.00)^a^	0.039*	5.78 (1.75, 19.12)^a^	0.004*
AST/ALT ratio	17.25 (0.30, >100.00)	0.168	1.26 (0.68, 2.33)	0.466
CPK in serum	1.00 (1.00, 1.00)	0.570	1.00 (1.00, 1.00)	0.365
Epileptic status	88.63 (0.27, >1,000.00)	0.128	1.00 (0.21, 4.78)	1.000
Liver lesions	0.00 (0.00, 20.07)	0.172	0.72 (0.18, 2.90)	0.644
Transfer to another department	0.01 (0.00, >1,000.00)	0.397	0.46 (0.05, 4.57)	0.509
According to year of admission	7.66 (0.52, 113.24)	0.139	1.28 (0.91, 1.80)	0.152

*p < 0.05 (significant difference); ^a^Clinical significance (OR > 3); AST/ALT, aspartate aminotransferase/alanine aminotransferase; CPK, creatine phosphokinase; OR, odds ratio; CI, confidence interval.

## Discussion

The psychiatric setting is not usually the location of choice for treating patients with DT. However, historically, DT has been considered as a consequence of alcoholism, and so all patients with DT are still primarily treated at the Psychiatric Clinic of the Clinical Center ‘Kragujevac’. In spite of that, mortality during the 10 years of our study was 7.37% which is similar to that recorded in other studies [2,3,6,9-11,24]. The lethal outcome was not registered during the last 3 years (2009–2011) for patients treated for DT in the Psychiatric Clinic.

Although the risk factors for DT are well recognized, their association with mortality is less well understood. Patients' age, comorbidities, duration of alcohol consumption, duration of treatment, serum potassium level, and history of epileptic seizures were identified as the most significant predictors of mortality in DT in previous research [2,6,17,19,21,23].

There are a number of studies which point to a drop in the serum potassium level during weaning from alcohol, especially when this is complicated further by DT [17,21,25-30]. Hypokalemia is actually a part of the complex adaptive mechanism, activated by the patient's body in order to overcome disturbances caused by a sudden lack of ethanol in the blood. The sympathetic system is activated, and consequently, blood levels of aldosterone and atrial natriuretic peptide (ANP) rise to very high levels [31,32]. The natriuretic effect of ANP is counterbalanced by the retention of sodium which is achieved by the aldosterone effects on renal tubules. The effect is maintenance of blood pressure and prevention of intracellular edema. However, the reabsorption of sodium by the renal tubules is accompanied by a loss of potassium into urine, as each sodium ion is exchanged for one potassium ion. Hence, high serum potassium levels in a patient with DT could indicate inappropriate adaptation to alcohol withdrawal and predict a fatal outcome in that patient [33].

Therefore, our observation that increased serum levels of potassium are associated with fatal outcome in DT patients is not surprising. Monitoring of serum levels of potassium is important not only as an indicator for replacement but also as an indicator of maladaptation. Patients with an increase in potassium (or absence of hypokalemia) may require more intensive treatment.

It is important to note that the average length of treatment for all cases was short (4.29 ± 3.77 days), and more than 60% of these patients died within the first 4 days following admission (four patients died on the day of admission, two on the second day, one on the third day, and two died on the fourth day). The length of treatment has also been found to be associated with mortality in previous studies [18,21]. It is clear that the adaptive mechanisms of these patients had been surpassed and that they required more intensive monitoring and treatment. In particular, the absence of adaptive hypokalemia was an early sign of maladaptation and should have indicated that these patients required more intensive treatment.

## Conclusions

The factors determining survival after admission for delirium tremens in the psychiatric setting depend on serum potassium levels, the number of hospitalizations, and duration of hospitalization. A significant association with lethal outcome was found only for serum potassium levels even in the normal range. Monitoring of serum levels of potassium is important not only as an indicator for replacement but also as an indicator of maladaptation. Patients with an increase in potassium (or absence of hypokalemia) may require more intensive treatment. The future researches are necessary in a larger sample of patients for better understanding of risk factors for lethal outcome in patients with delirium tremens.

### Limitations

The major limitation of the study relates to the small sample size due to low mortality over the study period. Also, some potentially predictive variables, such as sodium, chlorides, bicarbonates, blood urea nitrogen, ketones, magnesium, and pH, were not considered as this was a retrospective study and the necessary data had not been recorded. As a result, the findings of the study should be interpreted with caution and regarded as hypothesis-generating, in terms of predictive value of the absence of hypokalemia for fatal outcome in patients with DT.

## Abbreviations

ANP: Atrial natriuretic peptide; AST/ALT: Aspartate aminotransferase/alanine aminotransferase levels; CI: 95% confidence intervals; CPK: Creatine phosphokinase; DT: Delirium tremens; K+: Potassium in serum; M: Mean; OR: Crude and adjusted odds ratio; SDs: Standard deviations; t: Student's *t* test; U: Mann-Whitney test; %: Frequency of exposure; χ2: Chi-square test or Fisher's exact test.

## Competing interests

The authors wish to thank the Ministry of Education and Science of the Republic of Serbia for project nos. 175014 and 175007 from which the clinical investigation that served as the basis for this original work was partly financed.

## Authors’ contributions

DIR coordinated the teamwork of co-authors and wrote the paper. NR collected the data, searched for literature, and assisted with the writing of the article. SJ designed the study, supervised the data collection, and wrote the paper. SS was responsible for design of the study as well as for the statistical analysis in general. All authors read and approved the final manuscript.
